# Five novel RNA viruses of the invasive big-headed ant (*Pheidole megacephala*)

**DOI:** 10.1007/s00705-025-06375-6

**Published:** 2025-08-06

**Authors:** Charly T. Hartle, Chih-Chi Lee, Hung-Wei Hsu, Chun-Yi Lin, Kuan-Ling Liu, Joey Yin-Xin Chang, John A. Lawrence, Jia-Wei Tay, Chin-Cheng Scotty Yang

**Affiliations:** 1 Department of Entomology, Virginia Polytechnic Institute and State University, 24061 Blacksburg, VA USA; 2 Biotechnology Center in Southern Taiwan, Academia Sinica, 711010 Tainan, Taiwan; 3 Institute of Biomedical Sciences, Academia Sinica, 115201 Taipei, Taiwan; 4 Citrus Research and Education Center, University of Florida, 33850 Lake Alfred, Florida USA; 5 Department of Entomology, Texas Department of Entomology, Texas A&M University, 77843 College Station, Texas USA; 6 Department of Plant and Environmental Protection Sciences, University of Hawaii at Manoa, 96822 Honolulu, Hawaii USA

## Abstract

The African big-headed ant, *Pheidole megacephala*, is one of the most destructive invasive ant species, posing a significant threat to native biodiversity and ecosystem function, particularly in island ecosystems. Despite recent efforts to characterize pathogens in other major invasive ants, *P*. *megacephala* has received comparatively little attention. In this study, we characterized five novel RNA viruses actively replicating in *P*. *megacephala*, which we have named Pheidole megacephala virus 1–5 (PmV1-5), and examined viral diversity and prevalence in two invasive populations in Taiwan and Hawaii. This ant possesses a unique trait of true morphological worker dimorphism (major and minor), allowing us to test whether virus prevalence differs between the major and minor worker castes. Our findings indicate that all five novel viruses were present in both populations, but viral prevalence was higher in Hawaii than in Taiwan, potentially due to differences in supercolony structure. Multiple-virus infections were common, with coinfection by PmV1 and PmV5 being the most frequent. Notably, we found no significant difference in infection patterns between major and minor workers, suggesting that virus distribution is stable within colonies and that sampling different castes does not introduce bias in pathogen detection. This study represents the first characterization of viral pathogens in *P*. *megacephala* and may contribute to the development of microbial control strategies against this globally invasive ant.

**Supplementary Information:**

The online version contains supplementary material available at 10.1007/s00705-025-06375-6.

## Introduction

Human activity has facilitated the spread of invasive ants across the world, including the African big-headed ant, *Pheidole megacephala* [1, 2]. This ant species has a substantial negative impact on the native biodiversity of the areas in which it has been introduced [3–5], causing displacement of native ants, reductions in invertebrate and insectivorous bird populations, and extinction of multiple insect species [6, 7]. *Pheidole megacephala* is also known to impede biological control efforts and damage agricultural production by protecting and enhancing populations of plant-feeding hemipterans [8]. Furthermore, *P. megacephala* is a nuisance pest in residential areas, where it may feed on human food and damage wiring [5, 6]. In its invasive range, *P. megacephala* tends to form large, interconnected colonies with reduced aggression towards conspecifics while exhibiting high aggression towards other ant species [9]. These traits contribute to its competitive ability, allowing it to dominate an area and achieve much greater abundance than native ants despite its small body size [3].

The Enemy Release Hypothesis is one of the major theories explaining the success of invasive ants in their introduced environments, which is attributed to arriving without many of their natural enemies, including pathogens that could temper population expansion [10–12]. However, Yang *et al.* [11] have suggested that while the more detrimental, virulent pathogens may be filtered out during invasion, single-stranded RNA viruses (ssRNA) that cause asymptomatic infections may persist within the population and therefore tend to be co-introduced with the host. Some of the traits that allow invasive ants to dominate and thrive in introduced areas, such as high densities and unicoloniality, also contribute to horizontal virus transmission [13, 14]. However, a lack of colony boundaries and frequent interactions between colony members provide ample opportunity for pathogens to spread throughout the colony and population [14]. Populations of invasive ants such as the Argentine ant (*Linepithema humile*) and yellow crazy ant (*Anoplolepis gracilipes*) have flourished and then collapsed over time, with pathogens believed to be one possible cause [15–17].

The desire to control invasive ants has led to the discovery of multiple viruses in the red imported fire ant (*Solenopsis invicta*) [18], Argentine ant [19], and yellow crazy ant [20] using metatranscriptomic approaches. As of 2024, 66 viruses have been discovered to replicate in ants, the majority of which are positive-sense ssRNA (+ ssRNA) viruses belonging to the families *Dicistroviridae*, *Iflaviridae*, *Polycipiviridae*, and *Solinviviridae* of the order *Picornavirales* [21]. Although Brettell *et al.* [22] described several honeybee viruses infecting *P. megacephala* collected from Hawaiian apiaries, the prevalence and diversity of “ant” viruses infecting *P. megacephala* have yet to be described. The lack of studies examining the pathogens of *P. megacephala* is surprising considering the ant’s status as one of the five most destructive invasive ants in the world [23].

The genus *Pheidole* is distinct among the top genera of invasive ants due to the characteristic of true worker caste differentiation (body size dimorphism with no intermediate body size) into minor and major workers (hereafter, minors and majors), each with their own behavioral adaptations [24]. Majors specialize in defense, retrieval of larger food, and food storage and processing, whereas minors predominantly forage, care for brood, and also defend the colony [24, 25]. This system provides a unique opportunity to examine possible differences in virus transmission within the same ant species and colony due to behavioral differentiation in the worker castes.

Here, we characterized five novel + ssRNA viruses infecting *P. megacephala* and examined their prevalence and diversity in two invasive populations in Hawaii and Taiwan. We also compared the prevalence and diversity of these viruses between the two worker castes to examine whether viral infection patterns can be shaped by caste-specific behavior. Our data provide new insights into the distribution of pathogens between distinct invasive populations, the differentiation in virus prevalence among the worker caste, and the potential for the application of viruses as a widespread microbial biocontrol method for managing invasive *P. megacephala*.

## Materials and methods

### Collection and species identification

Two *P. megacephala* colonies were collected from the roadside in a semi-natural habitat in Okinawa, Japan (26°40’19.2”N, 128°00’41.0”E). The ants were identified at the species level using a combination of morphological identification following the methods of Bolton [26], Lin [27], and Sarnat *et al.* [28] and DNA analysis. One worker was sampled from each colony, and its DNA was extracted using a Gentra Puregene Tissue Kit (QIAGEN). A 708-bp fragment of the cytochrome oxidase I (COI) gene was targeted using the primers LCO1490 and HCO2198 [29], and polymerase chain reaction (PCR) was performed in a 25-µl reaction mixture containing 2 µl of template DNA, 12.5 µl of TaKaRa EmeraldAmp Max PCR Master Mix (TaKaRa), 0.2 µM each primer, and molecular-grade water. The reaction conditions were 94 °C for 3 min; 35 cycles of 94 °C for 30 s, 55 °C for 30 s, and 72 °C for 40 s; and 72 °C for 10 min. The amplified DNA was then purified using a Zymo DNA Clean and Concentrator-5 Kit (Zymo Research, Irvine, California, USA) and sequenced by the Sanger method. The mtDNA sequences were edited and aligned using ClustalW implemented in BioEdit [30] and MEGA v7.0 [31] and compared to COI sequences of *P. megacephala* reported previously by Liu *et al.* [29].

### RNA extraction and sequencing

Four worker individuals from each Okinawa colony were randomly selected to establish an RNA pool for RNA sequencing. The entire worker ant was soaked and homogenized with a pestle in TRIzol RNA Extraction Reagent (Invitrogen, Carlsbad, CA, USA), and the standard TRIzol RNA extraction protocol was followed. The RNA quality and quantity were measured using a NanoDrop spectrophotometer and then mixed at a 1:1 ratio based on the total RNA concentration of each sample. The pooled RNA was submitted to Eurofins Genomics (Tokyo, Japan) for sequencing after RNA purification using polyA selection. A sequencing library was constructed using a TruSeq Stranded mRNA Library Prep Kit (Illumina, San Diego, CA, USA) with an average insert size of 200 bp. The library was sequenced in paired-end mode (2 × 150 bp) using an Illumina (HiSeq 4000) platform.

### Viral genome sequence assembly and characterization

The paired-end reads obtained from Illumina sequencing were trimmed to remove adaptors and low-quality sequences (q < 30; base call accuracy < 99.9%), using Trimmomatic v0.39 [32]. The trimmed sequences were then assembled *de novo* using Trinity v2.11.0 [33] into transcripts, which were compared to the NCBI GenBank non-redundant protein database [34] using BLASTx [35] implemented in Diamond v2.0.11 [36]. Sequences with a high level of similarity to sequences from known viruses were selected using MEGAN Community Edition v6.18.8 [37] and examined using NCBI ORF Finder [34], NCBI Conserved Domains Search [34], and HHpred [38], using the Pfam databases [39] to identify conserved domains in the encoded proteins of each virus-like sequence. The pair-end reads were mapped onto Trinity contigs using Bowtie2 [40], and the transcripts per million (TPM) value was then calculated using RSEM [41]. The sequences were then compared to sequences in the NCBI nucleotide collection and non-redundant protein sequence databases [34] using BLASTn and BLASTx [35], respectively, to infer their taxonomy.

For further taxonomic classification of each virus, a viral proteomic tree based on genome-wide similarities was generated using ViPTree [42] with the BIONJ algorithm by inputting the whole genome sequences of the five newly identified viruses and comparing them to those of 4,415 eukaryotic ssRNA viruses obtained from Virus-Host DB [43]. The (unrooted) tree was then visualized using iTOL v5.0 [44]. Additionally, due to our discovery of two viruses of the family *Polycipiviridae*, pairwise comparisons of the amino acid sequences of the encoded RNA-dependent RNA polymerases (RdRps) were performed to determine whether they were distinct within our dataset.

### Virus prevalence

To determine the field prevalence of each novel virus of *P. megacephala*, minor worker ants were collected from 47 colonies in Hawaii and 25 colonies in Taiwan and preserved in ethanol (Supplementary Table S1). *Pheidole megacephala* exhibits distinct supercolony structures at these two locations [29], providing an excellent opportunity to test whether supercolony structure plays a role in shaping viral infection patterns (see Discussion for more details). High-quality RNA was extracted from a homogenized, pooled sample of 10 randomly selected adult minor worker ants from each of the 72 colonies using an E.Z.N.A. Total RNA Kit (Omega Biotek, Norcross, Georgia, USA). Virus prevalence was estimated based on minor worker data alone due to the limited number of colonies from which major workers could be sampled. To determine whether the viral infection pattern differed between the two castes, we also extracted RNA from a pooled sample of 1–4 adult major workers from colonies where major workers were collected (Hawaii, n = 18; Taiwan, n = 11).

Primers were designed for amplifying RdRp gene fragments of different lengths for each of the five novel viruses (Supplementary Table S2). cDNA was synthesized using random hexamer primers and a RevertAid RT Reverse Transcription Kit (Thermo Fisher Scientific, Waltham, Massachusetts, USA) and amplified by PCR using primers specific for the RdRp region (Supplementary Table S2), 2X Red Taq MasterMix (Apex Bioresearch Products, USA), and nuclease-free water. The reaction conditions were as follows: one cycle of 95 °C for 3 min, 35 cycles of 94 °C for 30 s, 54 °C for 30 s, and 72 °C for 30 s, and one cycle of 72 °C for 7 min. The presence of a target virus was confirmed by a single, clear band with the expected size on a 1% agarose gel. Samples were considered negative if bands were not clearly visible or not of the expected size.

To enable simultaneous detection of multiple viruses of *P. megacephala*, we developed a multiplex PCR protocol by using the aforementioned RdRp region primers, each specific for one of the five target viruses (Supplementary Table S2). Using cDNA synthesized with random hexamer primers as a template for PCR, we tested multiple combinations of our primer sets to determine which combination allowed all viruses to be amplified when present. Two multiplex PCR reactions, one with PmV2, PmV3, and PmV4 and the other with PmV1 and PmV5 (Supplementary Table S3), yielded the most robust amplification (i.e., the known viruses were amplified with comparable band intensities). For multiplex PCR, the thermal cycling profiles were the same as above, except the extension step at 72 °C was for 1 min rather than 30 s. The standard PCR and multiplex PCR gave identical detection results.

### Virus replication

We also examined whether the viruses were replicating in *P. megacephala* and therefore actively infecting the ants. The single-stranded RNA viruses identified in this study all have positive-sense genomes, and in order for them to replicate, a complimentary negative strand must be produced, which then serves as a template for generating more virus [45, 46]. The amplification of a negative strand therefore indicates that the virus is actively replicating inside the ant’s cells. For each virus, minor worker samples from four different Hawaii colonies of known virus status were tested for virus replication. Synthesis of cDNA was performed using a forward primer for each virus (Supplementary Table S2) tagged with the sequence 5’-AGCCTGCGCACCGTGG-3’ at 45 °C for 1 hour and 72 °C for 5 min. Touchdown RT-PCR was then performed using the strain-specific cDNA with the tag and reverse primers (Supplementary Table S2), 2X Red Taq MasterMix (Apex Bioresearch Products, USA), and nuclease-free water. The reaction conditions were one cycle of 95 °C for 3 min, 10 cycles of 94 °C for 30 s, 59 − 54 °C for 30 s (−0.5°C per cycle), and 72 °C for 1 min, followed by 25 cycles of 94 °C for 30 s, 50 °C for 30 s, and 72 °C for 1 min, and then one cycle of 72 °C for 7 min., and amplification was confirmed by 1% agarose gel as explained above.

### Statistical analysis

Statistical analysis was performed using R Statistical Software version 4.4.1 [47]. We constructed a contingency table for the viruses and colony location and performed a chi-squared test to test for associations. To determine whether the prevalence of the five novel viruses (together and each individually) was associated with colony location (Hawaii or Taiwan) or worker caste (minor or major), generalized linear mixed models (GLMM) were fitted using glmer in the lme4 package [48]. Binomial models were fitted for virus prevalence as a function of either colony location or caste and virus type, with virus presence as the dependent variable and either colony location or caste and virus type as the fixed effects. Colony ID was included as a random effect. All model assumptions were verified using the DHARMa package [49] and showed successful model convergence, normality of residuals, no overdispersion, no zero-inflation, low correlation between predictors, and no multicollinearity. *Post hoc* pairwise contrasts were performed on location (Hawaii or Taiwan) and caste (major or minor) using the emmeans package [50]. The VennDiagram package [51] was used to construct Venn diagrams.

## Results

### Ant species identification

Morphological identification and mitochondrial DNA analysis of the COI gene both confirmed the species identity as *P*. *megacephala.* All mitochondrial DNA sequences determined in this study were identical to haplotype TW1, a common *P*. *megacephala* mitochondrial haplotype [29].

### Virus discovery/characterization

**Fig. 1:**
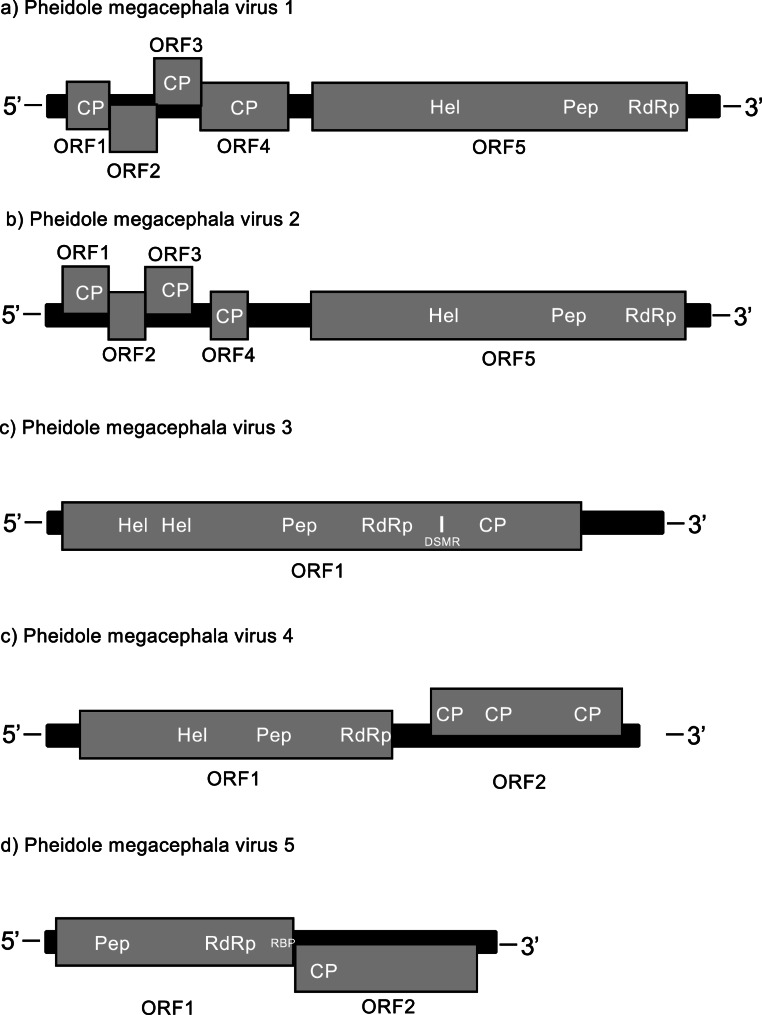
Genomic organization of five novel *Pheidole megacephala* viruses. ORFs are represented by grey rectangles with vertical offsets indicating reading frames (horizontal: frame 1, up: frame 2, down: frame 3) relative to the RdRp ORF (frame 1). Pep, peptidase; CP, coat protein; Hel, RNA helicase; DSMR, double-stranded RNA binding motif; RBP, RNA binding protein

**Fig. 2:**
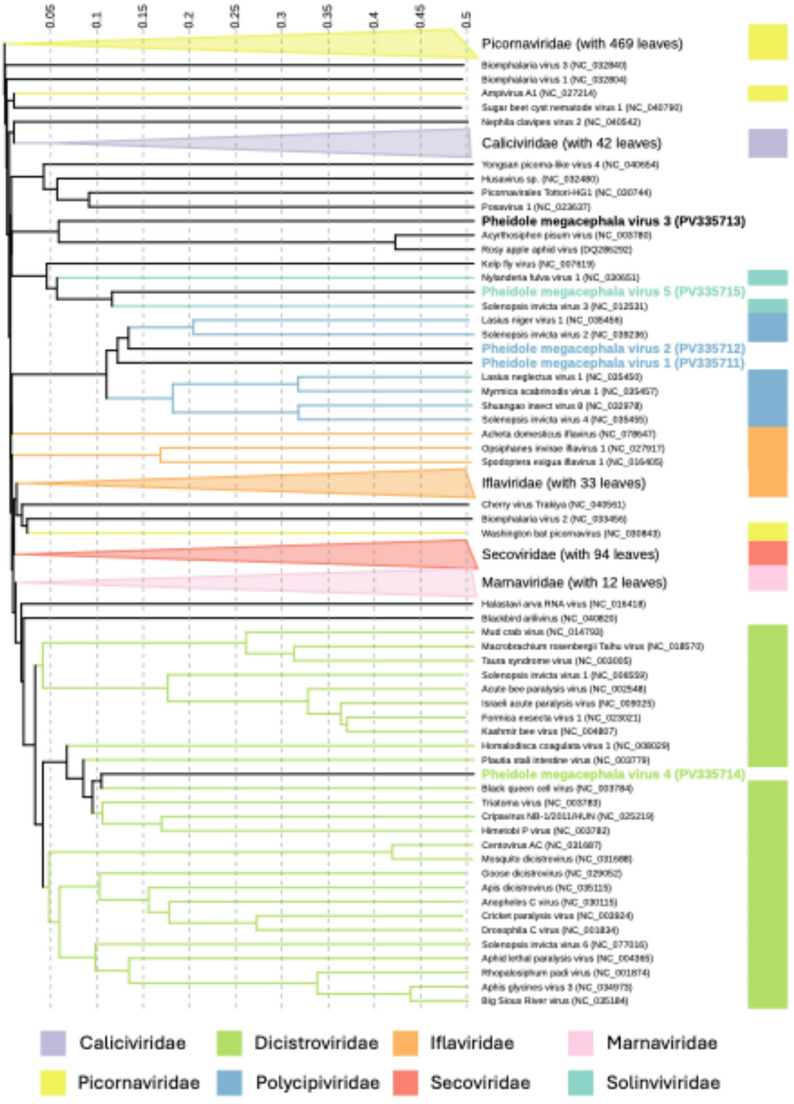
Viral proteomic tree of the newly discovered viruses from *Pheidole megacephala* and 704 related eukaryotic single-stranded RNA (ssRNA) viruses. For clarity, some clades were collapsed in the visualization. Colored bars and branches represent members of the order *Picornavirales*, with viral families assigned based on established taxonomy data from the Virus-Host DB. Colored labels indicate the inferred viral families of the Pheidole megacephala viruses identified in this study. Branch lengths are based on BIONJ-calculated genomic distances and are shown with linear scaling

Of the 2.6 million read pairs that passed the quality control (QC), MEGAN analysis identified 174 transcripts (including isoforms) as potential virus candidates. Of these, 19 transcripts (including isoforms) contained a complete RdRp conserved domain sequence, representing eight distinct potential viruses. The TPM from 1.49 to 9280.49 represented the abundance of viruses (Table 1, Supplementary Table S4). Of these transcripts, three showed more than 90% amino acid sequence identity to the RdRp region of viral sequences in the NCBI database (Supplementary Table S4). The remaining five transcripts were identified as possible novel viruses and were able to be assigned to a virus family based on sequence similarity in the RdRp region to known viruses in the NCBI database (Table 1). Two of these viruses were assigned to virus family *Polycipiviridae*, with genome lengths of 11,465 and 11,244 nt respectively, one was assigned to the order *Picornavirales*, with a of length of 10,495 nt, one was assigned to the family *Dicistroviridae*, with a length of 10,043 nt, and one was assigned to the family *Solinviviridae*, with a length of 7,672 nt (Table 1). In the order in which they are listed in Table 1, we tentatively named these novel viruses Pheidole megacephala virus 1, 2, 3, 4, and 5 (PmV1-V5; GenBank accession numbers PV335711 to PV335715). The viral genomes were annotated based on conserved features within their genomes (Table 2, Fig. 1). The genomic structures and features matched those described by the International Committee on Taxonomy of Viruses (ICTV) for the families *Polycipiviridae* [52], *Dicistroviridae* [53], and *Solinviviridae* [54]. A phylogenetic tree was constructed based on the five new virus sequences and 704 closely related eukaryotic ssRNA viruses (Fig. 2). This placed PmV5 in the same clade as Solenopsis invicta virus 3 and PmV4 in the same clade as black queen cell virus, while PmV3 is most closely related to two aphid-infecting RNA viruses and PmV1 and PmV2 are most closely related to other ant viruses, including Lasius niger virus 1 and Solenopsis invicta virus 2 (Fig. 2). A pairwise amino acid sequence comparison of the RdRp regions of the two novel *Polycipiviridae* viruses demonstrated that they were distinct within our dataset (Supplementary Table S5). The negative strand was successfully amplified for the five novel viruses from workers (in both Hawaii and Taiwan), indicating active replication by the viruses in *P. megacephala* (Supplementary Fig. S1).

**Table 1:** Genomic profiles of five novel viruses discovered in *Pheidole megacephala*

Virus	RdRp contained isoform	Length (nt)	Coverage (%)	TPM	Virus order/family	Top hit on NCBI	RdRp aa identity*
Pheidole megacephala virus 1	1	11,465	99.62	14.01	*Polycipiviridae*	Polycipiviridae sp.	72.51%
Pheidole megacephala virus 2	7	11,244	99.87	429.96	*Polycipiviridae*	Polycipiviridae sp., Electric ant polycipivirus 2	77.82%
Pheidole megacephala virus 3	2	10,495	100.00	9280.49	*Picornavirales*	Picornavirales sp., Apis picorna-like virus 5	64.06%, 61.57%
Pheidole megacephala virus 4	1	10,043	99.88	561.11	*Dicistroviridae*	Dicistroviridae sp.	67.35%
Pheidole megacephala virus 5	3	7,672	100.00	17.20	*Solinviviridae*	Hubei orthoptera virus 3	83.74%

*We compared the amino acid (aa) sequences of the RdRp conserved region of our virus-like transcripts to those of known viruses in the NCBI non-redundant protein database. Coverage (%) indicates the proportion of the viral genome with mapped reads

### Virus distribution/prevalence

**Fig. 3:**
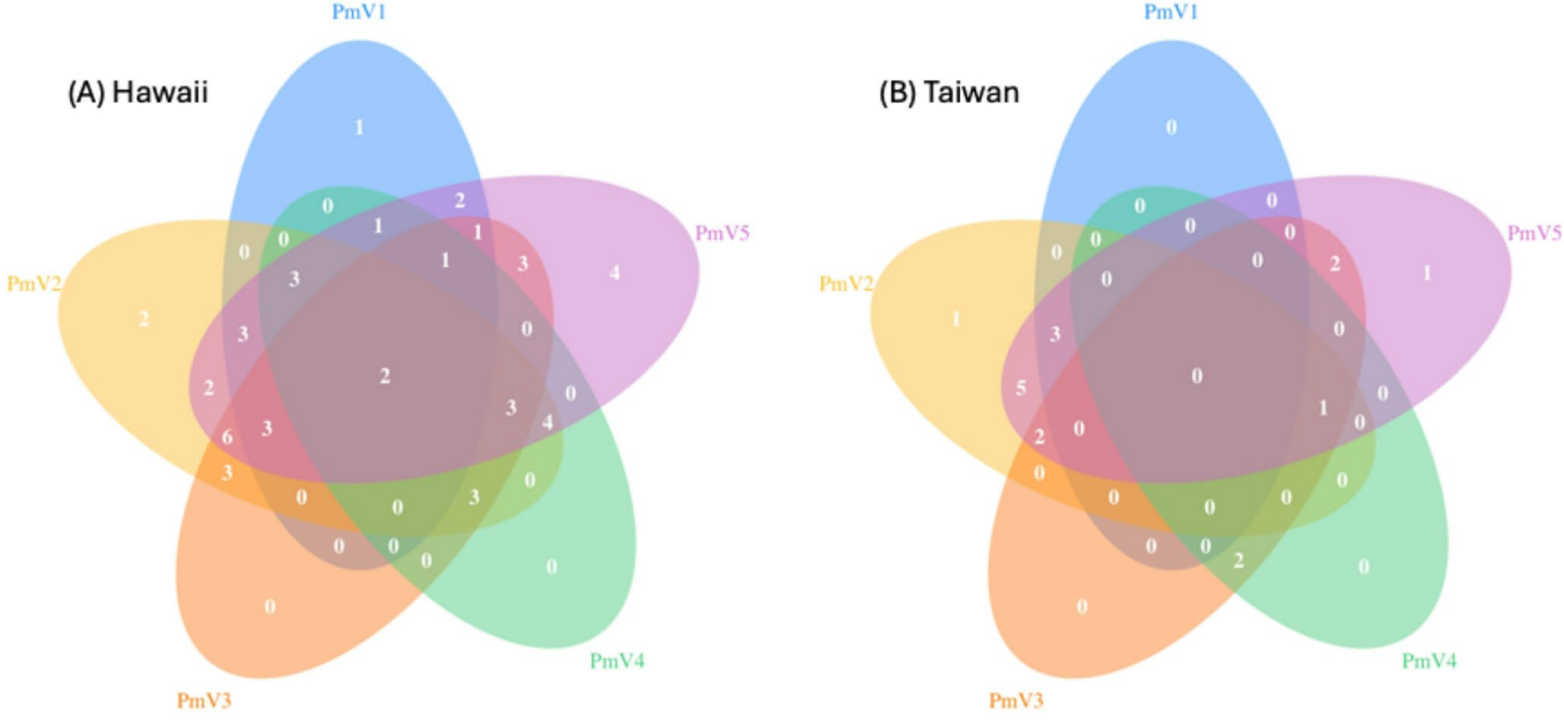
Virus occurrence in *Pheidole megacephala* colonies from the two sample locations: (**A**) Hawaii and (**B**) Taiwan. Each virus (PmV1-5) is represented by a different color, and numbers represent occurrence, either alone (no overlap) or together (overlapping ovals)

All five viruses were detected in both Hawaii and Taiwan, although their prevalence varied (Table 3). GLMM showed that the Hawaii and Taiwan colonies differed significantly in virus prevalence (*p* < 0.01). Every sampled colony from Hawaii was positive for at least one of the five viruses, whereas only 17 out of the 25 sampled colonies from Taiwan were infected. The likelihood of virus infection was found to be lower in the Taiwan colonies (GLMM estimate, 1.287, *p* < 0.01) compared to those in Hawaii. However, location alone was not a strong predictor of the number of viruses a colony may harbor (R2 = 19.95%). PmV5 was the most prevalent virus in both populations (Hawaii, 80.9%; Taiwan, 56.0%), while PmV1 and PmV4 were the least prevalent (36.17% and 12.00% for each respective population). Multi-infection, defined as the presence of two or more viruses within a colony, was common in both populations, with 85.1% of the Hawaii colonies and 60% of Taiwan colonies infected by at least two viruses (Fig. 3). PmV2 and PmV5 frequently co-occurred (with or without other viruses), coinfecting 24 out of 40 colonies across both locations. Interestingly, neither PmV3 nor PmV4 was detected as a single infection in any colonies from Hawaii or Taiwan. The prevalence of PmV1 and PmV5 differed significantly between the two locations (chi-square test; *p* < 0.01).

**Table 2:** Genomic structures and characterization of five novel RNA viruses from *Pheidole megacephala*

Virus	Length (nt)	Virus order/family	ORF	Conserved domain	Start position (nt)	End position (nt)	Frame	Tool/e-value
PmV1	11,465	*Polycipiviridae*						
			ORF1		291	1049	1	HPDB/9.3e-31
				Capsid	423	1041		
			ORF2		1046	1798	3	
			ORF3		1795	2634	2	
				Capsid	1936	2536		HPDB/7.3e-30
			ORF4		2631	4097	1	
				Capsid	3027	3555		HPDB/2.1e-22
			ORF5		4515	10907	1	
				RNA helicase	6603	6912		NCDS/1.59e-32
				Peptidase	8619	9279		HPfam/7.4e-17
				RdRp	9882	10818		NCDS/4.78e-175
PmV2	11,244	*Polycipiviridae*						
			ORF1		208	990	2	
				Capsid	379	982		HPDB/3.7e-34
			ORF2		987	1643	1	
			ORF3		1618	2439	2	
				Capsid	1756	2371		HPDB/2.2e-30
			ORF4		2436	3521	1	
				Capsid	2739	3393		HPDB/7.6e-23
			ORF5		4404	10847	1	
				RNA helicase	6483	6792		NCDS/9.5e-32
				Peptidase	8523	9180		HPfam/7.2e-17
				RdRp	9801	10752		NCDS/1.28e-162
PmV3	10,495	*Picornavirales*						
			ORF1		193	8964	1	
				RNA helicase	1219	1522		NCDS/1.78e-14
				RNA helicase	1972	2215		HPDB/3.2e-3
				Peptidase	3895	4525		HPfam/4.4e-15
				RdRp	5191	6040		NCDS/8.35e-35
				DSMR*	6550	6637		NCDS/5.08e-4
				Capsid	7078	7783		HPDB/1.7e-16
PmV4	10,043	*Dicistroviridae*						
			ORF1		553	5892	1	
				RNA helicase	2311	2665		NCDS/4.2e-30
				Peptidase	3559	4198		NCDS/6.99e-4
				RdRp	4864	5815		NCDS/1.47e-144
			ORF2		6530	9823	2	
				Capsid	6617	6833		NCDS/4.95e-4
				Capsid	7319	7835		NCDS/6.31e-19
				Capsid	8837	9476		NCDS/1.85e-21
PmV5	7,672	*Solinviviridae*						
			ORF1		187	4254	1	
				Peptidase	847	1531		HPfam/7.5e-18
				RdRp	2734	3685		NCDS/6.23e-75
				R2D2	4015	4225		HPfam/8.8e-3
			ORF2		4257	7373	3	
				Capsid	4371	4953		HPDB/3e-11

*Double-stranded RNA binding motif

NCDS: NCBI Conserved Domain Search against the CDD v3.21 database

HPDB: HHpred search against PDF database version PDB_mmCIF70_30_Mar

HPfam: HHpred search against Pfam database version Pfam-A_v37

Comparison of virus infection status showed that minor and major workers from the same colony generally shared a similar virus species composition (Table 3; Supplementary Table S1). Similarly, our statistical analysis failed to detect significant differences in virus prevalence between castes (GLMM; *p* = 0.562, Table 3). Although post-hoc pairwise contrasts showed a somewhat higher rate of virus occurrence in minor ants (4%), caste did not significantly affect virus status according to our model, and the “increase” may be due to the pooling of more minor workers (*N* = 10) for RNA extraction than major workers (*N* = 1–4).

## Discussion

Our study resulted in the discovery of five novel viruses in *P*. *megacephala*: PmV1, PmV2, PmV3, PmV4, and PmV5. Phylogenetic analysis placed PmV1 and PmV2 within the virus family *Polycipiviridae*, a common arthropod virus family with the majority of members infecting ants. For example, the red imported fire ant virus Solenopsis invicta virus 2 (SINV2), a gut-infecting polycipivirus, is horizontally transmittable through feeding but causes largely asymptomatic infections [55]. PmV3, classified within the order *Picornavirales*, clustered in a clade with unclassified aphid-infecting viruses, while PmV4 was placed within another common arthropod virus family, *Dicistroviridae*, which includes notable members such as Israeli acute paralysis virus (IAPV) and Solenopsis invicta virus 1 (SINV1), the last of which has been discussed for use as a biocontrol agent due to its potential to increase colony mortality in infected colonies with a high virus load or additional stress [56]. PmV5, the most prevalent virus in our samples, was placed within the family *Solinviviridae*, another family of arthropod viruses within the order *Picornavirales*. Two viruses within the family are known to infect ants [54], including Solenopsis invicta virus 3 (SINV3). Flynn and Moreau [57] profiled over 3,700 ant-associated DNA and RNA viruses and found that the most abundant ant-associated RNA viruses fell within the Picorna-Calici clade, which includes the virus families *Polycipiviridae* and *Dicistroviridae* and other unclassified *Picornavirales* viruses. Our results are concordant with the results of Flynn and Moreau [57] and others [12, 19, 58–60], supporting the conclusion that *Picornavirales* viruses, particularly polycipiviruses and dicistroviruses, are prevalent in ants.

**Table 3:** Prevalence (%) of five novel viruses in *Pheidole megacephala* colonies from Hawaii and Taiwan

	PmV1	PmV2	PmV3	PmV4	PmV5	Multi-infection	Total colonies infected
Location							
Hawaii	36.17	72.34	53.19	36.17	80.85	40/47	47/47
Taiwan	12.00	48.00	28.00	12.00	56.00	15/25	17/25
Caste							
Major	20.69	48.28	17.24	31.03	100.00	21/29	26/29
Minor	20.69	51.72	37.93	20.69	82.76	22/29	26/29

In addition to the five novel viruses described in this study, we detected three additional viruses whose encoded proteins showed high amino acid sequence similarity to those of previously published viruses, including human-blood-associated dicistrovirus (HBDV) [61], Orius laevigatus dicistrovirus 1 [62], and another virus belonging to the family *Dicistroviridae* [63] (Supplementary Table S4). Among them, HBDV, whose RdRp region shares > 98% sequence identity with our virus sequence (TRINITY_DN1919, Supplementary Table S4), was originally identified in human patients with fever symptoms [61]. Phan *et al.* [61] hypothesized an arthropod origin of HBDV but were unable to amplify the arthropod COI gene from their samples, leaving the possibility of arthropod DNA contamination unverified. However, in this study, negative-strand RNA of HBDV was detected by tagged RT-PCR in two *P. megacephala* samples (Supplementary Fig. S1, Supplementary Table S4), indicating active replication of this virus and supporting the possibility of its arthropod origin.

Our virus detection assays revealed that most of the viruses were prevalent in the Hawaii *P. megacephala* colonies surveyed, with every sampled colony infected by at least one virus. *Pheidole megacephala* is invasive in Hawaii, where it dominates the island’s ant communities. Lawrence *et al.* [unpublished data] found that, while *P. megacephala* is highly aggressive towards other ant species, it exhibits extremely low aggression toward conspecifics, even toward those from distant (45 km) nesting sites, suggesting that they comprise a single, large supercolony. Low intraspecific aggression allows frequent, close contact between worker ants from different colonies and may facilitate the horizontal virus transmission [14, 64]. In contrast, virus prevalence in the Taiwan *P. megacephala* colonies was lower in general, which may at least partially be explained by differences in colony boundaries. Liu *et al.* [29] tested the aggression between *P. megacephala* colonies in Taiwan and found intraspecific aggression between colonies separated by as little as 100 m. These colony boundaries may restrict virus spread by reducing contact between workers from different colonies, a pattern consistent with findings in other invasive ants (e.g., yellow crazy ants), where aggression has been shown to limit virus transmission between conspecifics [65]. Our data further support the “vulnerable supercolony hypothesis” [65–67], which predicts that ants with a supercolony structure are more susceptible to pathogen infections due to the high frequency of intercolonial interactions. We further predict that infection by viruses may be more widespread than those caused by other pathogens in supercolonial ants, as viruses are generally transmitted more readily through horizontal pathways [68, 69] and can evade certain immune defenses that ants use against other pathogens [70]. Our findings reinforce that viral infection patterns in ant populations are largely shaped by the interplay between viral transmission mode and social structure, which serve as predictors of virus diversity and prevalence.

Our study is the first to examine viruses in an ant species with true morphological worker dimorphism, where majors and minors perform distinct roles within the colony. Task differentiation between castes may lead to varying levels of contact within or between nests, potentially influencing virus transmission. However, our results indicate that caste was not a significant predictor of virus infection status in our samples. Given that RNA viruses in ants are highly transmissible and primarily spread through trophallaxis [12, 18], frequent social interactions between castes likely homogenize infection patterns within a colony. While some major workers specialize in food storage [25, 71], they may eventually share stored food with nestmates, including minor workers, further facilitating virus transmission and homogenizing virus diversity and prevalence across castes, resulting in a colony-level infection status that remains constant. As a result, sampling different castes within a colony is unlikely to affect assessments of viral infection status.

We found that multi-infections tended to be more common than single infections in the two studied *P. megacephala* populations. Major outbreaks of invasive ant populations on islands, including *P. megacephala* in Hawaii, have been followed by significant population declines [13], though other collapses may have happened unnoticed. While the mechanisms driving these fluctuations remain unclear, it is possible that viruses may contribute to population crashes, either directly, by reducing individual fitness, or indirectly, by exacerbating stress in dense colonies with reduced genetic diversity [13]. For example, red imported fire ants reach higher densities in their invaded areas compared to their native conspecifics [72] and often carry viruses that reduce fecundity [73] and lower foraging activity [74], potentially contributing to lowered fitness. Pathogens, including viruses, have been implicated in population crashes in several highly invasive species in multiple studies [75], and founder effects may cause invasive ants to have lower levels of immunity towards pathogens [76]. The “invasive species population extinction vortex model” posits that ants are introduced in small populations, which then expand into large, interconnected colonies. These high-density populations gain a competitive advantage but may ultimately collapse due to factors such as pathogen infections [13]. The highly dense populations of *P. megacephala*, which harbor numerous viruses, may be particularly vulnerable to collapse, although long-term monitoring data are necessary to test whether this hypothesis holds true.

Although the effects of the viruses described here in *P. megacephala* require further study, it is known that + ssRNA viruses often cause asymptomatic infections but can nevertheless be detrimental to a host that is under additional stress, where symptoms may become more acute [12]. There is evidence for a relationship between viral pathogenicity and prevalence in the host population in which highly virulent viruses are likely to induce stronger host immune responses, leading to their eventual elimination from the population, whereas less-virulent viruses may persist at higher prevalence [77]. This suggests that artificially augmenting these less-prevalent viruses could be a viable approach to biological control. Thus, developing methods to effectively distribute such pathogens to target ant colonies represents a key next step. Preliminary studies by Lawrence *et al.* [unpublished data] have shown the potential and feasibility of using hydrogel beads to deliver and disseminate viruses to *P. megacephala* in the field. Hydrogel beads are already established as an effective liquid bait delivery system for large-scale management of invasive ants [78, 79]. Combining liquid bait and virus delivery via hydrogel beads could synergistically stress ant colonies, potentially enhancing control efficacy.

## Electronic Supplementary Material

Below is the link to the electronic supplementary material

Supplementary Material 1

## Data Availability

All viral sequences in this article are publicly available (GenBank accession numbers PV335711-PV335715) and are also available in the supplementary material.
