# Supplementary material for: Five novel RNA viruses of the invasive big-headed ant (Pheidole megacephala)
Source: Arch Virol. 2025 Aug 6;170(9):190. doi: 10.1007/s00705-025-06375-6 (PMC12328527; doi:10.1007/s00705-025-06375-6)
Supplement: Supplementary file 1 — Supplementary Material 1 [file 705_2025_6375_MOESM1_ESM.docx]

**Supplemental Materials**

**Five novel RNA viruses of the invasive big-headed ant (*Pheidole megacephala*)**

Charly Hartle, Chih-Chi Lee, Hung-Wei Hsu, Chun-Yi Lin, Kuan-Ling Liu, Joey Yin Xin Chang, John A. Lawrence, Jia-Wei Tay, Chin-Cheng Scotty Yang

**Table S1** *Pheidole megacephala* sample metadata and virus status (N/A denotes that no major workers were collected from the given colony)

| Code | Location | Latitude, Longitude | Collection  Date | Virus  (Minors) | Virus  (Majors) |
| --- | --- | --- | --- | --- | --- |
| PM H01 | Hawaii | 21.063845, -157.803473 | 10/04/24 | PmV2, 3 | N/A |
| PM H02 | Hawaii | 21.291694, -157.806320 | 10/04/24 | PmV2 | N/A |
| PM H03 | Hawaii | 21.292850, -157.811193 | 10/04/24 | PmV2, 3 | N/A |
| PM H04 | Hawaii | 21.303351, -157.818848 | 10/04/24 | PmV2 | N/A |
| PM H05 | Hawaii | 21.315363, -157.810353 | 10/04/24 | PmV2, 3 | N/A |
| PM H06 | Hawaii | 21.315000, -157.807514 | 10/04/24 | PmV1 | N/A |
| PM H07 | Hawaii | 21.291718, -157.816516 | 02/26/24 | PmV2, 3, 4 | N/A |
| PM H08 | Hawaii | 21.291216, -157.816551 | 02/26/24 | PmV2, 3, 4 | N/A |
| PM H09 | Hawaii | 21.290883, -157.816352 | 02/26/24 | PmV2, 3, 4 | N/A |
| PM H10 | Hawaii | 21.267487, -157.786880 | 08/16/21 | PmV2, 5 | PmV2, 5 |
| PM H11 | Hawaii | 21.295630, -157.811446 | 08/16/21 | PmV5 | PmV5 |
| PM H12 | Hawaii | 21.301548, -157.816110 | 08/16/21 | PmV1, 2, 4, 5 | PmV1, 2, 4, 5 |
| PM H13 | Hawaii | 21.276646, -157.829800 | 08/16/21 | PmV3, 5 | PmV3, 5 |
| PM H14 | Hawaii | 21.317290, -157.801345 | 08/16/21 | PmV3, 5 | PmV3, 5 |
| PM H15 | Hawaii | 21.291135, -157.859059 | 08/16/21 | PmV1, 2, 3, 5 | N/A |
| PM H16 | Hawaii | 21.295446, -157.855492 | 08/16/21 | PmV1, 4, 5 | PmV1, 4, 5 |
| PM H17 | Hawaii | 21.401965, -158.005904 | 08/16/21 | PmV1, 2, 4, 5 | PmV1, 2, 5 |
| PM H18 | Hawaii | 21.269029, -157.819931 | 08/16/21 | PmV1, 2, 3, 4, 5 | N/A |
| PM H19 | Hawaii | 21.268863, -157.822462 | 08/31/21 | PmV3, 5 | PmV5 |
| PM H20 | Hawaii | 21.269291, -157.777397 | 08/31/21 | PmV2, 3, 5 | PmV2, 3, 5 |
| PM H21 | Hawaii | 21.293020, -157.850134 | 08/31/21 | PmV1, 5 | PmV1, 5 |
| PM H22 | Hawaii | 21.282737, -157.840014 | 08/31/21 | PmV5 | PmV5 |
| PM H23 | Hawaii | 21.314447, -157.862768 | 08/31/21 | PmV2, 4, 5 | PmV2, 4, 5 |
| PM H24 | Hawaii | 21.578291, -158.207783 | 08/31/21 | PmV2, 5 | N/A |
| PM H25 | Hawaii | 21.611056, -158.095522 | 09/14/21 | PmV2, 3, 4, 5 | PmV2, 4, 5 |
| PM H26 | Hawaii | 21.538646, -158.089518 | 09/14/21 | PmV2, 3, 5 | PmV2, 5 |
| PM H27 | Hawaii | 21.301290, -157.844857 | 09/14/21 | PmV1, 3, 4, 5 | N/A |
| PM H28 | Hawaii | 21.305125, -157.859821 | 09/14/21 | PmV2, 3, 5 | N/A |
| PM H29 | Hawaii | 21.300039, -157.816851 | 09/14/21 | PmV2, 3, 5 | PmV2, 5 |
| PM H30 | Hawaii | 21.291809, -157.678764 | 09/14/21 | PmV2, 3, 5 | PmV2, 5 |
| PM H31 | Hawaii | 21.395420, -157.724717 | 12/05/21 | PmV2, 4, 5 | PmV2, 4, 5 |
| PM H32 | Hawaii | 21.385804, -158.000315 | 12/05/21 | PmV1, 5 | PmV1, 4, 5 |
| PM H33 | Hawaii | 22.216314, -159.403920 | 12/05/21 | PmV5 | N/A |
| PM H34 | Hawaii | 21.303518, -157.818375 | 08/16/21 | PmV2, 4, 5 | N/A |
| PM H35 | Hawaii | 21.301485, -157.816210 | 08/16/21 | PmV2, 4, 5 | N/A |
| PM H36 | Hawaii | 21.293149, -157.811082 | 08/31/21 | PmV1, 2, 5 | N/A |
| PM H37 | Hawaii | 21.492209, -158.025986 | 09/14/21 | PmV1, 2, 3, 5 | N/A |
| PM H38 | Hawaii | 21.490887, -158.027182 | 09/14/21 | PmV1, 2, 3, 5 | N/A |
| PM H39 | Hawaii | 21.416630, -158.009285 | 09/15/21 | PmV2, 3, 4, 5 | N/A |
| PM H40 | Hawaii | 21.412858, -158.006117 | 09/15/21 | PmV1, 2, 4, 5 | N/A |
| PM H41 | Hawaii | 21.590696, -158.110055 | 12/05/21 | PmV1, 3, 5 | N/A |
| PM H42 | Hawaii | 21.592939, - 158.108882 | 12/05/21 | PmV1, 2, 5 | N/A |
| PM H43 | Hawaii | 21.660589, -157.927985 | 12/05/21 | PmV2, 3, 4, 5 | N/A |
| PM H44 | Hawaii | 21.575233, -158.123348 | 11/30/21 | PmV1, 2, 5 | N/A |
| PM H45 | Hawaii | 21.590696, -158.110055 | 11/30/21 | PmV1, 2, 3, 4, 5 | N/A |
| PM H46 | Hawaii | 21.592939, -158.108882 | 11/30/21 | PmV5 | N/A |
| PM H47 | Hawaii | 21.660589, -157.927985 | 11/30/21 | PmV2, 3, 5 | N/A |
| PM T01 | Taiwan | 25.099249, 121.534323 | 07/24/22 | PmV3, 5 | uninfected |
| PM T02 | Taiwan | 25.082667, 121.582990 | 07/24/22 | uninfected | N/A |
| PM T03 | Taiwan | 25.070323, 121.515758 | 07/24/22 | PmV2, 5 | N/A |
| PM T04 | Taiwan | 25.058625, 121.510334 | 07/24/22 | PmV2, 3, 4, 5 | N/A |
| PM T05 | Taiwan | 24.987073, 121.559944 | 08/17/22 | uninfected | uninfected |
| PM T06 | Taiwan | 25.032420, 121.534924 | 08/17/22 | uninfected | N/A |
| PM T07 | Taiwan | 24.118872, 120.677443 | 08/17/22 | PmV1, 2, 5 | PmV1, 2, 4, 5 |
| PM T08 | Taiwan | 24.148561, 120.730310 | 08/17/22 | PmV1, 2, 5 | N/A |
| PM T09 | Taiwan | 24.341050, 120.626001 | 08/17/22 | PmV5 | N/A |
| PM T10 | Taiwan | 24.350140, 120.624818 | 09/24/22 | uninfected | PmV3, 4 |
| PM T11 | Taiwan | 24.149892, 120.741127 | 09/24/22 | PmV3, 5 | PmV5 |
| PM T12 | Taiwan | 24.146398, 120.748707 | 09/24/22 | uninfected | N/A |
| PM T13 | Taiwan | 24.212496, 120.731028 | 09/24/22 | PmV1, 2, 5 | N/A |
| PM T14 | Taiwan | 22.682507, 120.300133 | 10/30/22 | uninfected | N/A |
| PM T15 | Taiwan | 22.646307, 120.305394 | 10/30/22 | PmV2, 5 | PmV5 |
| PM T16 | Taiwan | 22.649175, 120.294894 | 10/30/22 | PmV2, 5 | PmV2, 5 |
| PM T17 | Taiwan | 22.614296, 120.313852 | 10/30/22 | PmV2, 5 | N/A |
| PM T18 | Taiwan | 22.616242, 120.324176 | 10/30/22 | PmV2, 3, 5 | N/A |
| PM T19 | Taiwan | 22.639150, 120.286275 | 10/30/22 | PmV2 | uninfected |
| PM T20 | Taiwan | 22.631445, 120.293212 | 10/30/22 | uninfected | PmV3, 4 |
| PM T21 | Taiwan | 22.619787, 120.339560 | 10/30/22 | uninfected | N/A |
| PM T22 | Taiwan | 22.756942, 121.143593 | 10/30/22 | uninfected | N/A |
| PM T23 | Taiwan | 22.761696, 121.144555 | 10/30/22 | PmV2, 3, 5 | PmV2, 5 |
| PM T24 | Taiwan | 23.992730, 121.619320 | 10/30/22 | uninfected | PmV2, 5 |
| PM T25 | Taiwan | 23.971070, 121.594475 | 10/30/22 | uninfected | N/A |

**Table S2** Primers for virus detection and strain-specific RT-PCR

| **Virus** | **Primer code** | **Nucleotide sequence* (5’- 3’)** | **Amplicon size (bp)** |
| --- | --- | --- | --- |
| PmV1 | PmegV1_RdPp.F | CGGATTGGAGCAATGCTTAT | 490 |
|  | PmegV1_RdPp.R | CCAGTTTTGGAGGCTGATGT |  |
|  | Tagged forward V1 | agcctgcgcaccgtggCGGATTGGAGCAATGCTTAT |  |
| PmV2 | PmegV2_RdRp.F | TCCTCTCTGTCCCGGAATTA | 649 |
|  | PmegV2_RdRp.R | TAGCAAATCCAATGCGACTG |  |
|  | Tagged forward V2 | agcctgcgcaccgtggTCCTCTCTGTCCCGGAATTA |  |
| PmV3 | PmegV3_RdRp.F | CATGCCGCGGAACTATTTAT | 318 |
|  | PmegV3_RdRp.R | ATCGGGAGCAGAAATCAATG |  |
|  | Tagged forward V3 | agcctgcgcaccgtggCATGCCGCGGAACTATTTAT |  |
| PmV4 | PmegV4_RdRp.F | GCTCAAGGATGAGCGAAAAC | 207 |
|  | PmegV4_RdRp.R | TTCCGGTGCAATTCCTTTAC |  |
|  | Tagged forward V4 | agcctgcgcaccgtggGCTCAAGGATGAGCGAAAAC |  |
| PmV5 | PmegV5_RdRp.F | GCTGTACGATCACTGCTCCA | 248 |
|  | PmegV5_RdRp.R | TGCTGATGAATCAAGGGTCA |  |
|  | Tagged forward V5 | agcctgcgcaccgtggGCTGTACGATCACTGCTCCA |  |

* The annealing temperature for all primer sets is 54°C

**Table S3** Multiplex PCR reaction protocol

| **Multiplex** | **Virus** | **Primer code** | **Concentration** |
| --- | --- | --- | --- |
| Multiplex 1 PCR MasterMix (3.6 mM MgCl_2_) | PmV2 | PmegV2_RdRp.F | 0.2μL (10μM) |
|  |  | PmegV2_RdRp.R | 0.2μL (10μM) |
|  | PmV3 | PmegV3_RdRp.F | 0.2μL (10μM) |
|  |  | PmegV3_RdRp.R | 0.2μL (10μM) |
|  | PmV4 | PmegV4_RdRp.F | 0.3μL (10μM) |
|  |  | PmegV4_RdRp.R | 0.3μL (10μM) |
| Multiplex 2  PCR MasterMix (3.0 mM MgCl_2_) | PmV1 | PmegV1_RdRp.F | 0.2μL (10μM) |
|  |  | PmegV1_RdRp.R | 0.2μL (10μM) |
|  | PmV5 | PmegV5_RdRp.F | 0.1μL (10μM) |
|  |  | PmegV5_RdRp.R | 0.1μL (10μM) |

**Table S4** Similarity of three additional viruses assembled from *Pheidole megacephala* to known viruses

| Virus transcript ID | RdRp contained isoform | Length (nts) | TPM | Virus family | Top hit  on NCBI | GenBank accession#  [Reference] | RdRp aa identity* | Strain-specific  RT-PCR |
| --- | --- | --- | --- | --- | --- | --- | --- | --- |
| TRINITY_DN1919 | 3 | 6,713 | 2.33 | Dicistroviridae | Human blood-associated dicistrovirus | AWK23470  [64] | 98.94% | Yes |
| TRINITY_DN3368 | 1 | 6,097 | 1.49 | Dicistroviridae | Orius laevigatus dicistrovirus 1 | XGU09081  [65] | 99.28% | Yes |
| TRINITY_DN521 | 1 | 6,055 | 25.44 | Dicistroviridae | Dicistroviridae sp. | XCO49107  [66] | 94.90% | N/A |

* We compared the amino acid (aa) sequences of the RdRp conserved region of our virus-like transcripts to known viruses on NCBI’s non-redundant protein database.

**Table S5** Pairwise amino acid identity of RdRp region

| **Virus** | **PmV1** | **PmV2** | **PmV3** | **PmV4** |
| --- | --- | --- | --- | --- |
| **PmV2** | 55.06% |  |  |  |
| **PmV3** | 27.27% | 25.48% |  |  |
| **PmV4** | 24.14% | 22.22% | 27.89% |  |
| **PmV5** | 28.95% | 24.17% | 25.28% | 23.02% |


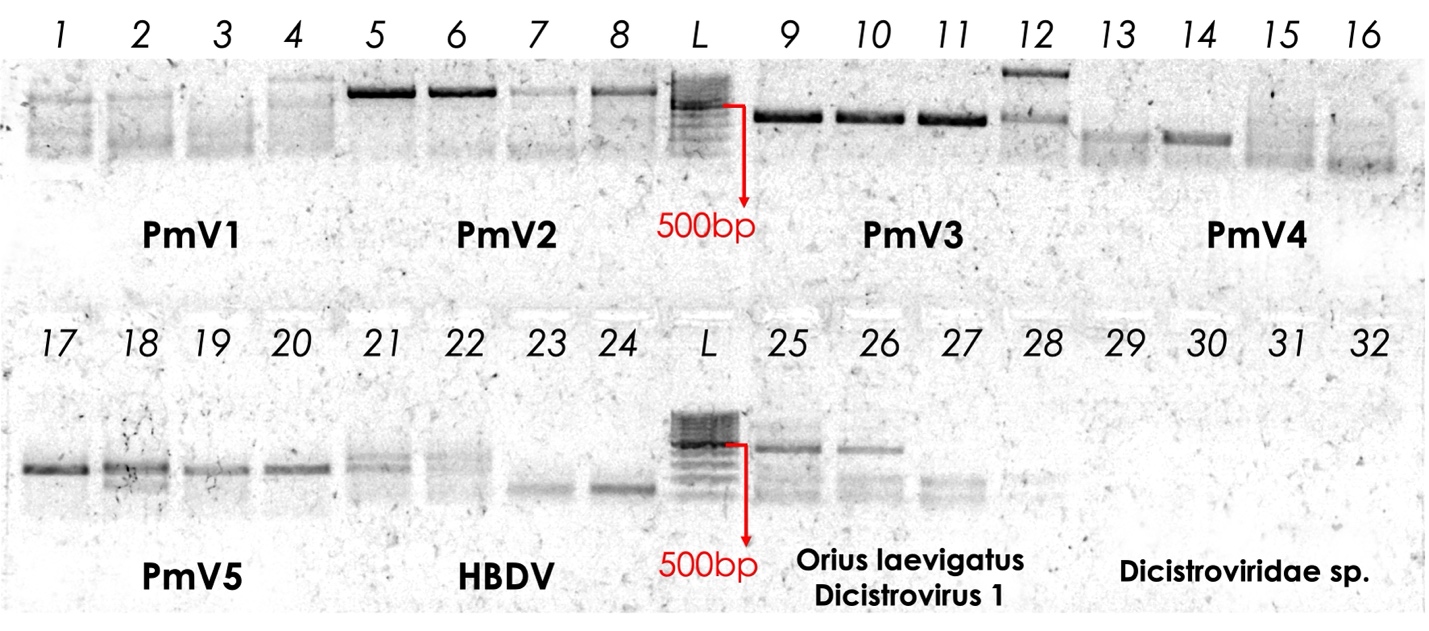


**Figure S1.** Amplification of the negative-strand RNA for each virus described in this study.

Lanes 1–4: PmV1; Lanes 5–8: PmV2; Lanes 9–12: PmV3; Lanes 13–16: PmV4; Lanes 17–20: PmV5; Lanes 21–24: HBDV (TRINITY_DN1919); Lanes 25–28: Orius laevigatus dicistrovirus 1 (TRINITY_DN3368); Lanes 29–32: Dicistroviridae sp (TRINITY_DN521). The expected amplicon sizes for HBDV, Orius laevigatus dicistrovirus 1, and Dicistroviridae sp. are approximately 333 bp, 550 bp, and 703 bp, respectively.
